# Roles of the ubiquitin proteasome system in the effects of drugs of abuse

**DOI:** 10.3389/fnmol.2014.00099

**Published:** 2015-01-06

**Authors:** Nicolas Massaly, Bernard Francès, Lionel Moulédous

**Affiliations:** ^1^Centre de Recherches sur la Cognition Animale, Centre National de la Recherche Scientifique UMR 5169Toulouse, France; ^2^Institut de Pharmacologie et de Biologie Structurale, Centre National de la Recherche Scientifique UMR 5089Toulouse, France; ^3^Université Paul Sabatier Toulouse IIIToulouse, France

**Keywords:** addiction, drug abuse, nicotine, opioid, plasticity, proteasome, stimulants, ubiquitin

## Abstract

Because of its ability to regulate the abundance of selected proteins the ubiquitin proteasome system (UPS) plays an important role in neuronal and synaptic plasticity. As a result various stages of learning and memory depend on UPS activity. Drug addiction, another phenomenon that relies on neuroplasticity, shares molecular substrates with memory processes. However, the necessity of proteasome-dependent protein degradation for the development of addiction has been poorly studied. Here we first review evidences from the literature that drugs of abuse regulate the expression and activity of the UPS system in the brain. We then provide a list of proteins which have been shown to be targeted to the proteasome following drug treatment and could thus be involved in neuronal adaptations underlying behaviors associated with drug use and abuse. Finally we describe the few studies that addressed the need for UPS-dependent protein degradation in animal models of addiction-related behaviors.

## The ubiquitin proteasome system (UPS)

**Figure 1:**
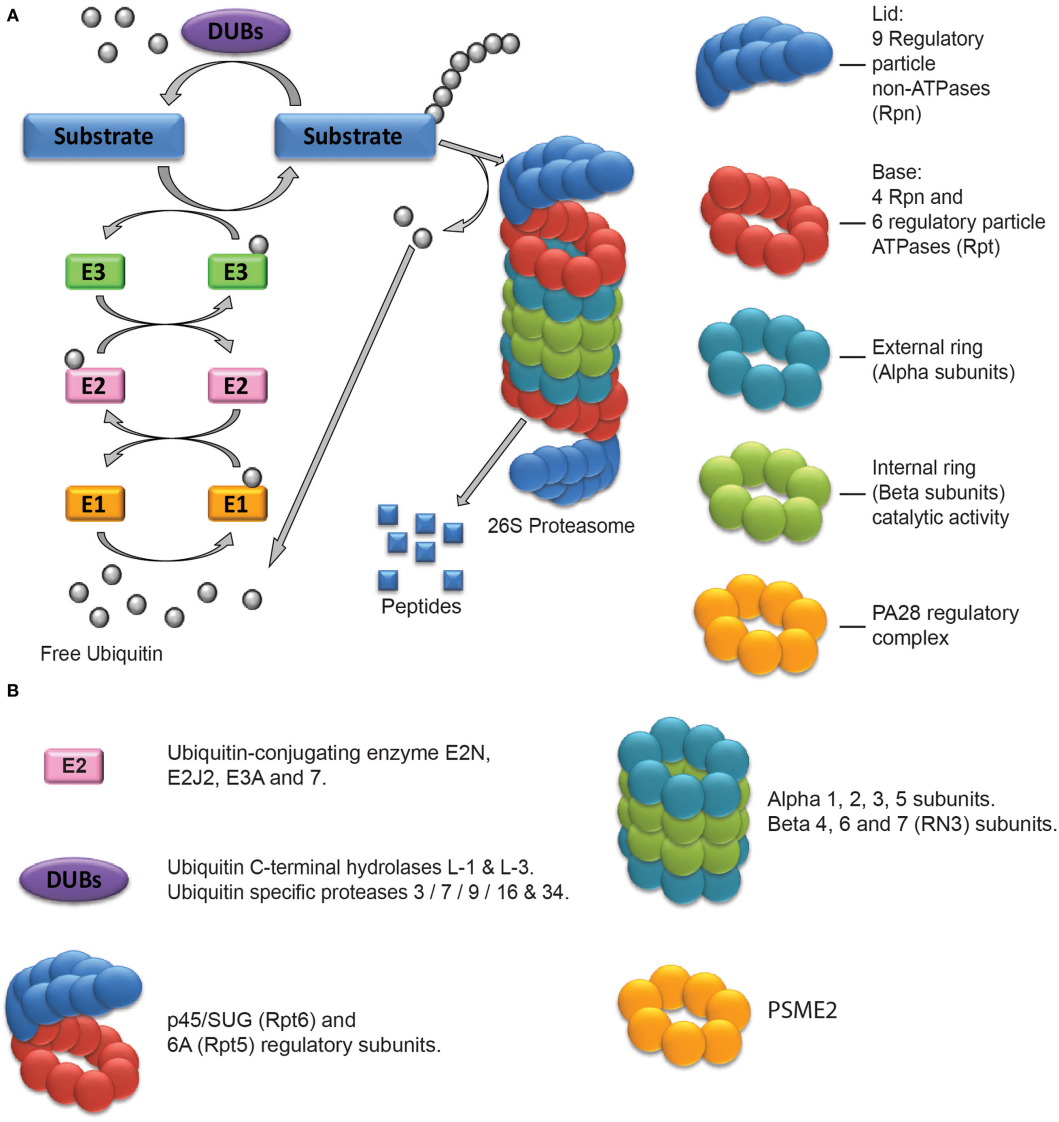
**The Ubiquitin Proteasome System and its components regulated after drug exposure. (A)** Schematic representation of the Ubiquitin Proteasome System. The external and internal rings constitute the 20S proteasome. The lid and base constitute the 19S regulatory complex. In some cases, it can be replaced by the PA28 or 11S regulatory complex, constituted of a single ring of 7 subunits. **(B)** Classification of the UPS components found to be regulated after drug exposure.

The role of protein turnover mediated by the ubiquitin proteasome system (UPS) in neuronal plasticity and memory has been studied for about two decades. Here we will only briefly summarize the basic functioning of this system that has been described in more detail in several reviews (Ciechanover, 2005; Patrick, 2006; Hegde, 2010; Mabb and Ehlers, 2010; Bingol and Sheng, 2011). The UPS controls the degradation of misfolded newly synthesized proteins as well as the turnover of specific target proteins. Its function can be described as a two-step process: the tagging of target proteins and their degradation. Ubiquitin molecules can be attached one to another and form a poly-ubiquitin chain which acts as a specific tag to direct proteins to proteasome-dependent degradation (Figure 1A). This enzymatic linkage is dependent on the activity of three types of enzymes: Ubiquitin-activating enzymes (E1), Ubiquitin-conjugating enzymes (E2), and Ubiquitin ligases (E3). E1 enzymes form a thioester bond with a ubiquitin molecule to activate it. The combined action of E2 and E3 enzymes then permits its linkage to a specific target protein. E3 enzymes mark the proteins that have to be degraded with a poly-ubiquitin chain (linked through Lysine 48 residues) but can also mediate mono- or other types of poly-ubiquinitation to affect different processes such as protein trafficking and kinase activation (see Bingol and Sheng, 2011 for a more detailed description). Another important class of enzymes is also involved in the regulation of poly-ubiquitination and UPS activity: the desubiquitinating enzymes (DUBs). They oppose the action of E3 ligases by removing ubiquitin. Thus, E1, E2, E3, and DUB enzymes tightly regulate the addressing of proteins to the proteasome. The second step of UPS function relies on the proteolytic activity of the 26S proteasome. This complex of proteins can be sub-divided into two components: the 20S proteasome which is the catalytic core where degradation takes place and the 19S proteasome which acts as a regulatory complex. The 20S proteasome is made of two external and two internal rings of proteins. External rings are composed of seven alpha type proteins (numbered from α1 to α7). They are involved in the regulation of the access of tagged proteins to the inner core (internal rings) of the 20S proteasome. The internal rings are composed of seven beta type proteins (numbered from β1 to β7) which are responsible for the catalytic activity of the proteasome. Three subunits are directly involved in degradation processes: the β1, β2, and β5 subunits which are responsible for caspase-like, trypsin-like and chymotrypsin-like activity respectively. The other types of β subunits have been proposed to play a structural role in the complex and to be involved in the binding of targeted proteins during their degradation by β1, β2, and β5 subunits. Different complexes can be associated with the 20S proteasome, the 19S proteasome being the most frequent. The 26S proteasome possesses two 19S proteasome regulatory complexes located at each extremity of the 20S core. They can also be divided in two distinct subparts: the lid and the base. The lid is composed by 9 regulatory particle non-ATPase (Rpn) proteins and possesses two main roles: the recognition of poly-ubiquitinated proteins and the removal of ubiquitin from the targeted proteins. The base is composed of 10 proteins with or without ATPase activity, Regulatory particle ATPases (Rpt) and Rpn proteins respectively. It is physically connected to the proteasome 20S and is involved in the unfolding of proteins and the regulation of their entry into the catalytic core. Thanks to the combined actions of E1, E2, E3, and DUB enzymes and the 19S proteasome complex, the UPS can finely control the identity of the proteins to be targeted and degraded by the catalytic core located in the inner part of the 20S proteasome.

## The UPS in neuronal plasticity and memory

Changes in neuronal activity can result in the regulation of many proteins by the UPS. A descriptive study showed that increases or decreases in the activity of cultured hippocampal neurons produce UPS-dependent changes in the amount of several proteins in post-synaptic densities (PSD), including proteins involved in PSD morphology, cytoskeleton organization and scaffolding of signaling complexes (Ehlers, 2003). This result suggests a close relationship between synaptic plasticity and protein degradation. Indeed, it has been reported that protein degradation by the UPS contributes to the formation and maintenance of long-term potentiation (LTP) and long-term depression (LTD). The first study reporting this involvement was conducted in Aplysia during induction of long-term facilitation (LTF) (Hegde et al., 1993). It demonstrated that regulatory subunits of the Protein kinase A (PKA) are targeted to the proteasome for degradation allowing prolonged action of PKA and Aplysia behavioral sensitization (Hegde et al., 1993). Later on, studies in rodents have shown that blocking the UPS in the hippocampus can alter N-methyl-D-aspartate (NMDA)- and/or metabotropic glutamate receptor (mGluR)-dependent LTD or LTP (Colledge et al., 2003; Citri et al., 2009). This deleterious effect of UPS blockade on long term changes in neurons has been suggested to be due to an alteration in the balance between protein synthesis and degradation (Fonseca et al., 2006). Indeed the authors showed that the deleterious effects produced by inhibiting either protein synthesis or degradation on LTP can be reversed by inhibition of the two processes at the same time. In addition to synaptic proteins the UPS is also involved in the regulation of the activity of transcription factors, thus revealing a close relationship between protein synthesis and proteasome action. For example IκB and CREM (cAMP-responsive element modulator), repressors of the transcription factors NF-κB and CREB (cAMP response element binding) respectively, can be ubiquitinated and degraded by the UPS (Woo et al., 2010; Liu and Chen, 2011). In that sense the UPS clearly plays a major role in the regulation of protein turnover implicated in neuronal plasticity acting directly through the degradation of some proteins and indirectly through the modulation of transcriptional activity and protein synthesis.

Unsurprisingly considering its role in neuronal plasticity, a strong involvement of UPS function has also been observed during learning and memory processes. These results are reported in detail in a recent review (Jarome and Helmstetter, 2014). More than 10 years ago a first study demonstrated the role of the proteasome in the dorsal hippocampus during the acquisition phase of inhibitory avoidance memory (Lopez-Salon et al., 2001). In this work the authors reported an increase in the rate of protein poly-ubiquitination in the hippocampus during training. They also found that the repressor of NF-κB, IκB, was present in this poly-ubiquitinated protein pool showing an involvement of the UPS in transcription factor activation. More recent studies confirmed the necessity of protein degradation in the hippocampus during consolidation and reconsolidation processes in rodents in spatial memory and fear conditioning (Artinian et al., 2008; Lee et al., 2008). The hippocampus is not the only region were proteasome activity is required for the creation and maintenance of memory. The involvement of protein degradation in both the prefrontal cortex and the amygdala during fear learning has also been demonstrated (Jarome et al., 2011; Reis et al., 2013). Proteasome action also appears to be necessary in both the insular cortex and the amygdala for aversive taste learning (Rodriguez-Ortiz et al., 2011).

The precise mechanisms underlying the involvement of the proteasome in memory are just beginning to be discovered but it is now clearly established that, in addition to protein synthesis, neuronal protein degradation by the UPS is a mandatory process to create, store and maintain memories and in that sense participates to adaptive behaviors of mammals. Since drug addiction shares common mechanisms with memory processes (Hyman et al., 2006; Milton and Everitt, 2012) it is important to question the role of the UPS in the long term effects of drugs of abuse such as opioids, stimulants, ethanol, nicotine and cannabinoids.

## Drugs of abuse regulate the UPS

In recent years, many transcriptomic and proteomic studies have described the global effects of treatments with drugs of abuse on the brain, or on neuronal or glial cell lines. Proteasome subunits or proteins involved in the ubiquitination process are often found to be regulated in these studies (Table 1, Figure 1B). In the case of opioids, it was shown in a cellular model that a prolonged 72 h morphine treatment modifies the abundance of two proteasome subunits (α3 and β6) (Neasta et al., 2006). *In vivo*, intra-cerebro-ventricular (icv) infusion of morphine for 72 h results in an increase in the tyrosine-phosphorylated form of the β4 subunit in the rat frontal cerebral cortex (Kim et al., 2005). A longer intermittent treatment (2 weeks) produces a decrease in the amount of the DUB Ubiquitin C-terminal hydrolase L-1 in the nucleus accumbens (Nacc) (Li et al., 2006). 4 days after morphine withdrawal, the quantity of this enzyme, as well as that of the α3 subunit of the proteasome, increases in rat dorsal root ganglia (Li et al., 2009). Similarly, chronic treatment (90 days) and drug withdrawal have been shown to have opposite effects on the amount of α5 subunit in the Nacc of rhesus monkeys (Bu et al., 2012). The levels of Ubiquitin-conjugating enzyme E2 and of Ubiquitin C-terminal hydrolase L-3 are also modulated in this model. Finally, in a morphine-induced conditioned place preference (CPP) paradigm which tests the rewarding properties of the drug, both development, extinction and re-instatement are accompanied by a down-regulation of several DUBs and α and β subunits (Lin et al., 2011).

**Table 1:** **UPS-related molecular and cellular consequences of the treatment with drugs of abuse**.

**Drug class**	**Drug**	**Treatment**	**Cell type/Species**	**Molecular/cellular effects**	**Reference**
Opioids	Morphine	72 h	Recombinant SH-SY5Y cells	Change in α3 and β6 subunit abundance	Neasta et al., 2006
	Morphine	24 h	Recombinant SH-SY5Y cells	UPS-dependent down-regulation of Gβ subunits of heterotrimeric	Mouledous et al., 2005
				G proteins	
	DAMGO	Overnight	Human SH-SY5Y cells	UPS-dependent down-regulation of RGS4	Wang and Traynor, 2011
	DADLE	40 min	Recombinant HEK cells	MOP receptor ubiquitination	Hislop et al., 2011
	Basal turnover	N.A.	Recombinant NMB cells	MOP receptor degradation by the UPS	Song et al., 2009
	Morphine	4 h and 24 h	Human SK-N-SM cells	Modulation of proteasome catalytic activity	Rambhia et al., 2005
	Morphine	48 h	Rat C6 glioma cells	UPS-dependent down-regulation of EAAC1 glutamate transporter	Yang et al., 2008b
	Morphine	7 days intra-thecal	Rat spinal cord	UPS-dependent down-regulation of EAAC1, GLAST, and GLT-1	Yang et al., 2008a
				glutamate transporters	
	Morphine	72 h icv	Rat frontal cortex	Increase in Tyr-phosphorylated β4 subunit	Kim et al., 2005
	Morphine	2 weeks	Rat nucleus accumbens	Decrease in Ubiquitin C-terminal hydrolase L-1	Li et al., 2006
	Morphine	CPP	Rat amygdala	Decrease in α3, α6, β3, β4, β7 subunits, Ubiquitin C-terminal	Lin et al., 2011
				hydrolase L-1 and Ubiquitin specific protease 7	
	Morphine	4 days after withdrawal	Rat dorsal root ganglia	Increase in Ubiquitin C-terminal hydrolase L-1 and α3 subunit	Li et al., 2009
	Morphine	4 days, increasing doses	Mouse striatum	Reduced UPS-dependent degradation of HSP70	Yang et al., 2014
	Morphine	CPP	Mouse Nacc synaptosomes	Increase in total protein ubiquitination	Massaly et al., 2013
	Morphine	90 days	Rhesus monkey Nacc	Increase in α5 subunit and decrease in Ubiquitin conjugating enzyme E2	Bu et al., 2012
	Morphine	Withdrawal	Rhesus monkey Nacc	Decrease in α5 subunit and increase in Ubiquitin C-terminal hydrolase L-3	Bu et al., 2012
Stimulants	Methamphetamine	3–18 h	N27 dopaminergic cells	Impaired proteasome activity	Lin et al., 2012
	Methamphetamine	Acute injection	Rat striatum	Increase in Ubiquitin C-terminal hydrolase L-1 and decrease in RN3 subunit	Iwazaki et al., 2006
	Methamphetamine	24–48 h	Rat striatum and frontal cortex	Transient decrease in 26S proteasome activity	Dietrich et al., 2005
	Methamphetamine	8 days	Rat frontal cortex	Increase in Ubiquitin C-terminal hydrolase L-1 and decrease	Faure et al., 2009
				in α1, α2 and regulatory 6A subunits	
	Amphetamine	7 days + withdrawal	Rat striatum PSD	UPS-dependent degradation of Shank and GKAP	Mao et al., 2009
	Cocaine	CPP	Rat medial prefrontal cortex	Increase in Ubiquitin-conjugating enzyme E2N, α2 and regulatory p45/SUG subunit	Guan and Guan, 2013
	Cocaine	24–48 h	Rat striatum and frontal cortex	Transient increase in 26S proteasome activity	Dietrich et al., 2005
	Cocaine	CPP	Rat Nacc core	UPS-dependent degradation of NSF protein	Ren et al., 2013
Ethanol	Ethanol	5 days	Mouse cortical neurons	Decrease in the mRNA of Ubiquitin-conjugating enzymes E2 and E3A, Ubiquitin specific protease 9, and 7 regulatory or catalytic subunits	Gutala et al., 2004
	Ethanol	4 months, drinking water	Mouse cortex	Impairment of UPS activity associated with an increase in immunoproteasome subunits	Pla et al., 2014
Nicotine	Nicotine	17 h	HEK cells, rat cortical neurons	Reduced ERAD-dependent degradation of α4β2 nicotinic acetycholine receptors	Govind et al., 2012
	Nicotine	8 h	Recombinant HELA cells	Reduced ERAD-dependent degradation of α3β4 nicotinic acetycholine receptors	Mazzo et al., 2013
	Nicotine	14 days	Rat prefrontal cortex	Increase in the mRNA of several Ubiquitin-conjugating enzymes, Ubiquitin proteases, and regulatory and catalytic subunits of the proteasome	Kane et al., 2004
	Nicotine	14 days	Rat medial basal hypothalamus	Decrease in the mRNA of several Ubiquitin-conjugating enzymes and α subunits	Kane et al., 2004
	Nicotine	14 days	Mouse dopaminergic neurons	Increase in the mRNA of the E2 ubiquitin-conjugating enzyme E2J2, decrease in that of PSME2 regulatory subunit and Ubiquitin specific proteases 16 and 34	Henley et al., 2013
	Nicotine	24 h	Mouse prefrontal cortex	Inhibition of UPS associated with increased glutamate receptor subunits and PSD95	Rezvani et al., 2007
Cannabinoids	Δ^9^-THC	48 h	Human astrocytes	Increase in the mRNA of Ubiquitin specific protease 3	Bindukumar et al., 2008
	HU-210	16 h	Neuro-2A cells	UPS-dependent degradation of Rap1GAPII resulting in neurite outgrowth	Jordan et al., 2005

Changes in expression of proteins of the UPS are not specific to opioid treatment. The amount of Ubiquitin C-terminal hydrolase L-1 is increased and that of RN3 (β7 catalytic subunit) is decreased in the striatum of rats acutely treated with methamphetamine while repeated injections induce an increase in Ubiquitin C-terminal hydrolase L-1 and a decrease in several proteasome subunits in the frontal cortex (Iwazaki et al., 2006; Faure et al., 2009; Kobeissy et al., 2009). The development of cocaine CPP comes with an increase in the expression of the Ubiquitin-conjugating enzyme E2N, of the catalytic α2 subunit and of the 26S proteasome regulatory subunit p45/SUG (Guan and Guan, 2013). Moreover, mouse cortical neurons grown in the presence of ethanol for 5 days show decreased amounts of mRNA coding for several ubiquitin-conjugating enzymes, as well as catalytic and regulatory subunits of the proteasome (Gutala et al., 2004) while the quantities of Ubiquitin C-terminal hydrolase L-1 decrease and that of ubiquitin and Ubiquitin-conjugating enzyme 7 increase in the white matter of the brain of alcoholic patients (Alexander-Kaufman et al., 2006; Kashem et al., 2007). Again at the mRNA level, chronic treatment of rats with nicotine produces an elevated expression of ubiquitin-conjugating enzymes, proteasome regulatory and catalytic subunits and DUBs in the prefrontal cortex whereas their level is decreased in the medial basal hypothalamus (Kane et al., 2004). Variations can be of opposite direction within a single cell type with for example an up-regulation of the E2 ubiquitin-conjugating enzyme E2J2 associated with the down-regulation of a proteasome regulatory subunit and two DUBs in mouse dopaminergic neurons chronically treated with nicotine (Henley et al., 2013). Finally an up-regulation of the DUB Ubiquitin specific protease 3 was observed in human astrocytes exposed for 48 h to Δ^9^-THC (tetra-hydro-cannabinol) (Bindukumar et al., 2008).

All drugs of abuse can thus affect the expression and abundance of key UPS proteins. However, the data reported above are only descriptive. Moreover, UPS components are affected differently depending on the drug type, its method of administration, the duration of the treatment and the cell type or brain region considered (Table 1). Complementary studies have also found that drugs of abuse modify the activity of the UPS in parallel with changes in the expression of its various components. Indeed morphine was demonstrated to inhibit the activity of the 20S proteasome in human neuroblastoma cells, with neuroprotective consequences (Rambhia et al., 2005). On the contrary, PKC-dependent inhibition of the UPS was linked to the autophagy-mediated toxicity of methamphetamine in dopaminergic neurons (Lin et al., 2012). In addition it has been proposed that the higher toxicity of methamphetamine compared to cocaine was due to its long inhibitory effect on proteasome activity (Dietrich et al., 2005). Finally, a recent study demonstrated that chronic ethanol induces toxicity in mice through a Toll-like receptor 4-dependent impairment of the UPS (Pla et al., 2014). This deleterious effect could depend on a shift in proteasome composition from classical to immunoproteasome subunits, a phenomenon known to play a role in the neurotoxicity observed in neurodegenerative diseases, and on an increase in chymotrypsin-like and trypsin-like activities (Pla et al., 2014).

So far we have only described global changes in the composition and/or activity of the UPS associated with beneficial or deleterious effects on the functioning or survival of neurons. However, more subtle and finely regulated mechanisms need to be considered to explain the plasticity phenomena underlying the development of addiction-related behaviors. These mechanisms do not necessarily imply a global modification of UPS activity but rather the degradation of specific targets in precise cellular locations. Unfortunately fewer studies have focused more specifically on synaptic and/or signaling proteins degraded by the UPS in relation with the administration of drugs of abuse.

## UPS targets involved in drug-induced plasticity

Drugs of abuse target receptors, channels and transporters located in the plasma membrane. However, membrane proteins are not typical proteasome substrates but are rather degraded in lysosomes. Proteasome-mediated degradation only occurs for misfolded membrane proteins through the ERAD (Endoplasmic-reticulum-associated protein degradation) pathway before their export to the plasma membrane (Christianson and Ye, 2014) but ubiquitination can also modulate the degradation of membrane proteins after endocytosis by influencing their sorting to lysosomes through the ESCRT (endosomal sorting complexes required for transport) system (Macgurn et al., 2012). This phenomenon involves HECT (Homologous to the E6-AP Carboxyl Terminus) E3 ligases and will not be discussed in detail here since it is proteasome-independent. However, it is worth mentioning that, since proteasome inhibitors cause the accumulation of ubiquitinated proteins and thus reduce the available pool of free ubiquitin, they can affect indirectly ubiquitin-dependent proteasome-independent processes such as sorting to lysosome (Mimnaugh et al., 1997).

Mu opioid (MOP) receptors play a role in the rewarding and reinforcing properties of opioids but also of most non-opioid abused drugs (Le Merrer et al., 2009). They are ubiquitinated following activation. Proteasome inhibitors increase their basal abundance and decrease agonist-induced down-regulation in recombinant cells (Chaturvedi et al., 2001). The increase in basal receptor expression following proteasome inhibition could be due to the blocking of the ERAD pathway whereas the reduction in agonist-induced down-regulation could result from the indirect effect of proteasome inhibitors on ubiquitin-dependent sorting to lysosomes. Indeed it was recently shown that the ubiquitination of the first intracellular loop of the MOP receptor facilitates its lysosomial degradation by promoting its transfer to intralumenal vesicles downstream of the ESCRT system (Hislop et al., 2011). It was also proposed that different translational forms of the receptor showed different sensitivities to the ERAD pathway because of additional ubiquitination sites (Song et al., 2009). Besides the MOP receptor, the nicotinic receptor is another example of drug target which has been shown to be regulated by the UPS. Here again the ERAD pathway seems to be involved and the subunit composition of pentameric nicotinic receptors has an influence on their sensitivity to this pathway (Govind et al., 2012; Mazzo et al., 2013).

UPS-dependent changes have also been identified downstream of receptor activation. In SH-SY5Y human neuroblastoma cells, long-term morphine treatment induces proteasome-dependent degradation of the Gβ subunit of heterotrimeric G proteins (Mouledous et al., 2005). This degradation could reduce G protein-coupled receptor signaling and restore the activity of effectors normally inhibited by Gβ subunits such as adenylyl cyclase. In the same cells, opioids have also been shown to induce the ubiquitination and degradation of regulator of G protein signaling 4 (RGS4), a protein that controls the duration of G protein signaling by acting as a GTPase accelerating protein (GAP) (Wang and Traynor, 2011). RGS4 is an unstable protein known to be subjected to the N-end rule pathway, a particular type of regulation based on the removal of the N-terminal methionine and the arginylation of the resulting N-terminal cysteine to promote ubiquitination and proteasome degradation. Its down-regulation affects the signaling of other G protein-coupled receptors present in the same cell. Overall, by regulating the abundance of several signaling molecules sensitive to opioid treatment, the UPS participates in the homeostatic processes involved in the development of opioid tolerance and dependence (Bailey and Connor, 2005; Christie, 2008). In mice, chronic morphine treatment induces a decrease in the total amount of ubiquitinated proteins in the striatum. In parallel, the heat-shock protein HSP70 was shown to be overexpressed, probably because of a lower ubiquitination rate (Yang et al., 2014). The higher expression of this protein could participate in the behavioral sensitization induced by morphine (Qin et al., 2013). However, the HSP70 cellular effect mediating this process is currently unknown. Besides changes in signaling, long-term drug treatment is known to affect neuronal structural plasticity (Robinson and Kolb, 2004). Small G proteins can influence cellular architecture and it is thus significant to note that, in neuro-2A cells, cannabinoids induce neurite outgrowth by activating the small G protein Rap1 through the proteasome-dependent degradation of one of its GAP, Rap1GAPII (Jordan et al., 2005).

The neuronal adaptations described so far are homeostatic non-associative phenomena. They result from the direct activation of the drug target and its downstream signaling and are not sufficient to explain the associative processes involved in addiction. Similarly to classical forms of memory, drug addiction involves activity-dependent plasticity at excitatory synapses within neuronal circuits, notably those controlling motivated behaviors (Kauer and Malenka, 2007; Russo et al., 2010). Following drug administration, the UPS system regulates the abundance of several proteins at the glutamatergic synapse but very few studies have identified these proteasome targets. In the case of opioids, an increase in ubiquitinated proteins in the synaptosomal fraction of the mouse Nacc was observed following morphine conditioning (Massaly et al., 2013) but the identity of the UPS targeted proteins was not reported. So far, the only glutamate-related proteins shown to be degraded by the proteasome following chronic morphine treatment are glutamate transporters EAAC1, GLSAT, and GLT-1 but these changes were observed in the rat spinal cord and were related to analgesic tolerance rather than addiction (Yang et al., 2008a,b). Concerning nicotine, one study addressed the effect of its intra-peritoneal injection in mice on the expression of synaptic proteins (Rezvani et al., 2007). It suggested that the observed increase in the amount of GluR1 α-amino-3-hydroxy-5-methyl-4-isoxazolepropionic acid (AMPA) receptor subunits, NR2A NMDA receptor subunits, metabotropic receptor mGluR1α, and PSD95 (a scaffolding protein of the PSD), but also the decrease in the quantity of Shank (another scaffolding protein), were due to an inhibition of proteasome activity. The exposure to stimulants was also reported to have UPS-dependent synaptic effects. NAC1 (nucleus accumbens-associated protein 1), the product of an immediate early gene up-regulated by psychostimulants, takes part in the recruitment of the proteasome to the PSD by interacting with Cullin-based E3 ubiquitin ligases and the 19S ATPase subunit Mov34 (Shen et al., 2007). The UPS could also contribute to the phenomenon of synaptic scaling that is observed in the Nacc following cocaine withdrawal and results from the addition of AMPA receptors to the synapse (Sun and Wolf, 2009). UPS-dependent synaptic changes in the striatum have been shown to contribute to the behavioral sensitization induced by repeated amphetamine injections in rats. Contrarily to acute nicotine injection, chronic amphetamine treatment produced a decrease in NMDA receptor subunits and anchoring proteins in the PSD. Only Shank and GKAP (guanylate-kinase-associated protein) were ubiquitinated and it was proposed that the degradation of these important anchoring proteins indirectly leads to a loss of PSD95 and NR1 and NR2B subunits of the NMDA receptor at the synapse (Mao et al., 2009). Finally, the retrieval of cocaine place preference in rats has been shown to result in an increase in protein poly-ubiquitination in the core of the Nacc, and in particular in the degradation of NSF (N-ethylmaleimide-sensitive fusion), a protein of the PSD involved in synaptic plasticity (Ren et al., 2013). In conclusion, the UPS is involved in the synaptic plasticity that underlies some of the behavioral adaptations to drug exposure. However, the molecular details are still poorly known and will probably depend on the drug type, the location of the synapse in the neuronal circuit and the phase of the addiction process under study. It is thus critical to implement studies to establish direct causal relationship between the degradation of neuronal proteins by the UPS in a particular brain region and a given addiction-related behavior.

## UPS and addiction-related behaviors

**Figure 2:**
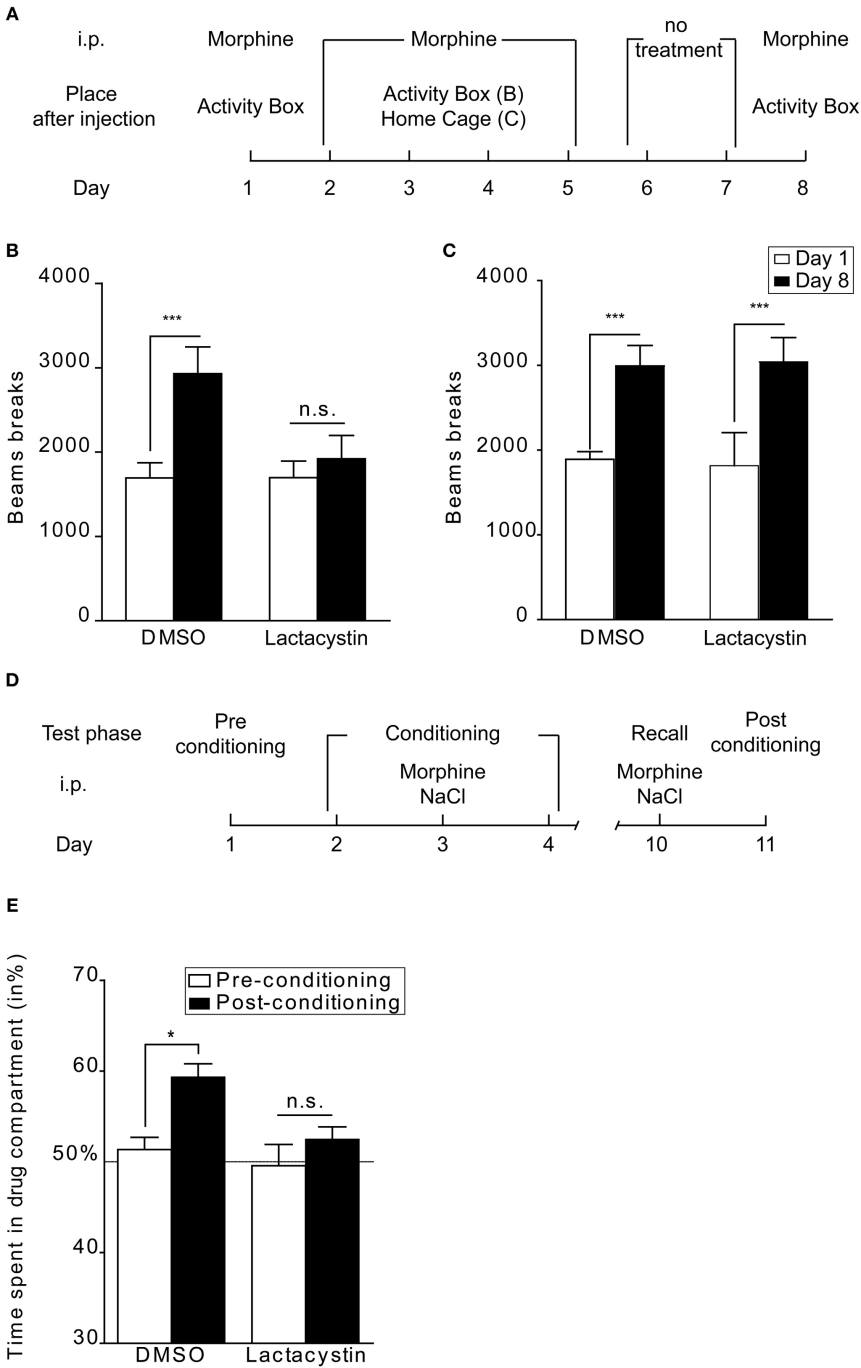
**UPS involvement in behavioral sensitization and reconsolidation of morphine place preference**. **(A)** Schematic representation of the protocol followed in context-dependent and -independent locomotor sensitization. The morphine dose was 10 mg/kg. **(B)** UPS inhibition blocks the acquisition of behavioral sensitization when a context-dependent paradigm is used (lactacystin 100 pmol in 0.5 μl per side: *n* = 8 and DMSO: *n* = 11), **(C)** whereas it does not affect this drug-adaptation in a context-independent procedure (lactacystin: *n* = 6 and DMSO: *n* = 8). Data are expressed in number of beam breaks ± SEM during a 1 h session after morphine injection before (day 1; empty bars) and after conditioning (day 8; black bars). Two-Way ANOVA followed by Bonferroni *post-hoc* tests: n.s., non-significant; ^***^p < 0.001. **(D)** Schematic representation of the protocol used for assessing the role of the UPS in reconsolidation. **(E)** Intra-Nacc bilateral injection of lactacystin 1 h before a drug-context re-exposure abolishes drug-induced place preference when tested 24 h after this new association (*n* = 6) whereas DMSO treated-animals still express place preference (*n* = 6). Data are expressed as percentage of time spent in the drug-associated compartment ± SEM during pre-conditioning tests (empty bars) and post-conditioning tests (filled bars). Two-Way ANOVA followed by Bonferroni *post-hoc* tests: n.s., non-significant; ^*^p < 0.05. See Massaly et al. (2013) for details about behavioral procedures.

Few studies have assessed the role of protein degradation by the proteasome in drug-related behaviors. Recently we found that UPS function in the Nacc is crucial in several types of opioid-induced behaviors (Massaly et al., 2013). Our goal was to assess the role of protein degradation by the proteasome in the development of drug seeking behaviors and the motivation to obtain opioids. By using proteasome inhibitors our study demonstrated a clear role of the UPS in the Nacc during acquisition of non-operant tasks, namely CPP and context-dependent locomotor sensitization in mice. Intra-Nacc proteasome inhibitors also prevented the acquisition of operant tasks in mice (intra-VTA self-administration) and rats (intra-venous self-administration). However, these behavioral paradigms do not enable us to clearly discriminate between an effect of proteasome inhibitors on drug-induced memory and on non-associative drug effects. The behavioral sensitization procedure can be implemented in a context-dependent or -independent way (Valjent et al., 2006). Figures 2A–C shows the comparison between the effects of proteasome inhibition in a context-dependent (Massaly et al., 2013) and a context-independent paradigm. In both experiments mice were submitted to 5 daily morphine injections followed by a 2 day withdrawal period. On day 1 basal horizontal activity was measured during 1 h directly after i.p. morphine injection (Figures 2B,C, empty bars). On days 2, 3, 4, and 5 mice received intra-Nacc injection of DMSO or the proteasome inhibitor lactacystin 1 h prior to i.p. morphine treatment and were then directly placed in their home cage to prevent association between drug and cues present in activity boxes (context-independent, Figure 2C) or in activity boxes (context-dependent, Figure 2B). Three days after the last opioid treatment, animals were challenged with an i.p. morphine injection and locomotor activity was measured during 1 h to assess behavioral sensitization. Control groups showed a significant locomotor sensitization on day 8 (Figures 1C, 2B, DMSO group). Lactacystin injections prevented behavioral sensitization only in the context-dependent procedure (Figure 2B). It thus appears that the UPS in the Nacc is not necessary for the development of behavioral sensitization when this adaptation is context-independent (Figures 2A–C, refer to (Massaly et al., 2013) for details). This result, together with the fact that intra-Nacc injection of proteasome inhibitors prevents the consolidation of morphine place preference, strongly suggests that UPS activity in the Nacc is involved in drug-context association rather than non-associative motivational effects of opioids. However, this distinction might not be true for each type of drug of abuse and in particular for stimulants. Indeed, proteasome inhibitors have been shown to inhibit the development of behavioral sensitization to amphetamine after intra-Nacc injection in rats in a context-independent paradigm (Mao et al., 2009).

Consolidation is the process by which stable long-term memories are formed following a learning session. Reconsolidation refers to the fact that the memory trace can return to an active labile state after recall (Alberini and Ledoux, 2013). Interfering with this process could thus offer a way to erase drug-context association with important therapeutic consequences. If lactacystin is injected in the Nacc before a new drug-compartment association performed 1 week after the last morphine place preference conditioning (Massaly et al., 2013, Figure 2D), mice do not display any place preference for the morphine compartment on the following day, contrarily to the DMSO control group (Figure 2E). The UPS in the Nacc thus seems to be involved in reconsolidation of place preference induced by morphine. Our conclusions concerning the involvement of the UPS in consolidation and reconsolidation of drug-associated memories are only partly consistent with those of the only other study examining this question (Ren et al., 2013). Ren et al. found that inhibiting UPS activity in the Nacc interfered with drug-reward memories using cocaine CPP in rats. However, in their model, proteasome inhibitors blocked CPP extinction when injected following each extinction session but were not efficient on memory consolidation during the learning phase. Moreover, they did not interfere directly with memory reconsolidation following a reactivation session but counteracted the inhibitory effect of protein synthesis inhibition on this process. These apparent discrepancies are likely due to many differences in experimental conditions between the two studies: animal model (rats vs. mice), drug type (cocaine vs. morphine), conditioning procedure (4 drug/saline injections over 8 days vs. 3 injections over 3 days), timing of injection of proteasome inhibitors (before vs. after memory reactivation), method of induction of memory reconsolidation (reactivation in the absence vs. in the presence of drug). Taken together, the two studies confirm that the UPS in the Nacc plays a key role in drug-reward memories although further work is needed to fully understand under which exact circumstances it is recruited.

In conclusion, even if some discrepancies can be observed between studies depending on the model under investigation, it appears that the UPS plays a role in drug-related behaviors and the adaptation to the exposure to drugs of abuse. Future work will certainly bring us new evidence to complete the picture of the involvement of proteasome-dependent protein degradation in the brain during addiction.

## Future directions

It is clear from the studies reviewed here that the UPS plays an essential role in neuronal plasticity associated with long-term exposure to drugs of abuse. However, the UPS is involved in so many cellular processes that we are still a long way from understanding its specific contribution to each aspect of drug use and abuse. Several outstanding questions need to be addressed to achieve this goal. What are the most significant cellular targets of the UPS during neuronal plasticity associated with drug addiction? So far, studies have focused on synaptic and signaling proteins but other types of proteins, such as for example transcription factors, are mandatory for enduring neuronal plasticity and could be regulated directly or indirectly by UPS-dependent processes (Carle et al., 2007; Dong et al., 2008). Where does the regulation take place? All the behavioral studies reported here focused on the Nacc but different UPS-dependent changes will probably occur depending on the brain region. Also alterations in protein content will vary according to the type of plasticity occurring in each individual neuron or synapse. When does the protein need to be degraded? Different UPS targets will be concerned depending on the phase of the addiction process. For example changes appearing along the course of drug administration will probably differ from those resulting from withdrawal. Why is a UPS-dependent regulation occurring? In particular it will be important to distinguish homeostatic regulations involved in non-associative tolerance or sensitization from more integrated plasticity phenomena responsible for associative context-dependent aspects of addiction. How are the proteins targeted to the proteasome? Identifying the mechanism by which each important UPS substrate is targeted to the proteasome (post-translational modification preceding ubiquitination, type of E3 ligase…) will offer opportunities to control addiction-related processes more specifically than by blocking the catalytic activity of the proteasome. Many technical limitations may prevent us from answering fully to these questions but the link between the control of protein expression through UPS-dependent degradation and plastic changes involved in addiction clearly deserves further investigation.

### Conflict of interest statement

The authors declare that the research was conducted in the absence of any commercial or financial relationships that could be construed as a potential conflict of interest.
